# Documentation of ethnoveterinary knowledge and alternative practices for cattle tick control in Sekhukhune District, Limpopo Province, South Africa

**DOI:** 10.3389/fvets.2024.1488960

**Published:** 2025-01-07

**Authors:** Confidence Semakane Phaahla, Jeremiah Leshweni Shai, Vincent Maduna, Resoketswe Charlotte Moropeng, Solomon Ramagoai Magano

**Affiliations:** ^1^Department of Life and Consumer Sciences, Faculty of Agriculture and Life Sciences, University of South Africa, Pretoria, South Africa; ^2^Department of Biomedical Sciences, Faculty of Science, Tshwane University of Technology, Pretoria, South Africa

**Keywords:** ethnoveterinary knowledge, indigenous communities, herbal acaricides, ticks, Sekhukhune District

## Abstract

**Introduction:**

The integration of traditional plant-based methods for controlling ectoparasites in the primary healthcare of livestock is progressively emerging as a crucial intervention to enhance livestock productivity in regions with limited resources, particularly in smallholder farming areas facing resource constraints. In Sekhukhune District, where livestock plays a vital role in rural livelihoods, cattle ticks present a significant challenge to cattle farming. This study aimed to document the ethnoveterinary practices employed by local communities to control cattle ticks, highlighting the use of alternative methods rooted in indigenous knowledge (IK).

**Methods:**

Data were collected using a purposive sampling method to select traditional livestock keepers, herders, and community elders to uncover the plant-based treatments and management strategies used in tick control. In addition, a semi-structured questionnaire and a guided field survey were employed to collect data.

**Results:**

A total of 250 participants, with an age range from 18 to over 60 years, were recruited. The result revealed that the elder participants, over 60 years of age, were more knowledgeable compared to the youth and adults. Furthermore, 28 plant species with potential acaricidal properties and other methods aimed at controlling cattle tick infestations were documented. *Cissus quadrangularis* was the most frequently cited species, which was widely distributed throughout the district.

**Conclusion:**

In addition, these results are framed within the larger context of sustainability, promoting eco-friendly cattle farming practices in Sekhukhune District while reducing reliance on conventional acaricides. By documenting this ethnoveterinary knowledge, the study contributes to the preservation of indigenous knowledge while advocating for sustainable approaches to livestock health management in rural areas. The study concludes with valuable insights into the selected local community’s traditional methods of managing tick infestations. Furthermore, the study underscores the significance of preserving and understanding indigenous knowledge in livestock health management, particularly in regions where conventional veterinary approaches may face challenges.

## Introduction

Indigenous knowledge (IK) is defined as a systematic body of knowledge developed by local individuals through an accumulation of informal experiments, beliefs, and understanding of their environment. It evolves through an adaptive process and is passed down through generations orally (1–5). In addition, the practice of IK differs across localities and cultural groups, each possessing different systems. Thus, these practices create a distinctive indigenous knowledge system (IKS). As the IKS is multi-cultural and dynamic, it allows for the continuous addition of new wisdom to the existing body of knowledge. As the exchange of the IKS occurs, so do the beliefs and procedures. However, the adaption of new practices is required to maintain a positive impact on the environment (6). Furthermore, the IKS is an important tool for local people as it is integral to the social structures of primary healthcare. Moreover, its contribution to livelihoods in developing countries cannot be undervalued as it is reported that approximately 80% of people in developing countries depend on traditional herbal mixtures to treat different diseases (7).

Indigenous knowledge systems (IKS) are widespread practices that are explored across the world. Furthermore, it has been discovered that selected medicinal plants used to treat human ailments can also be applied in primary healthcare for domestic animals, depending on local knowledge and the availability of natural resources (8). This knowledge could help bridge gaps in treating ailments commonly affecting livestock and distress its production. Recently, scientific reports have shown interest in documenting the IKS and exploring the bioactivities of medicinal plants for ethnoveterinary practices, similar to ethnobotany (9, 10) highlighting the influence of ethnoveterinary practices in rural areas.

Furthermore, ethnoveterinary medicine (EVM) continues to be practiced in developing countries, especially where public veterinary services are considered inefficient and financially unfeasible (11–16). The continued use of EVM is encouraged by the belief that traditional medicine is more effective than conventional medicine, with the presumption that it has no side effects as animals consume selected plants. These treatments are considered environmentally friendly (7, 12, 17, 18).

However, the risk of losing such significant IK may arise due to migration, urbanization, and modern technologies. These factors can interfere with the traditional means of knowledge transfer, leading to knowledge decline or misinformation. This has stimulated researchers to attempt to preserve the fragility of oral knowledge. In Limpopo Province, studies have been conducted on preserving plant species with acaricidal activity (12, 18–22).

Moreover, the studies mentioned above listed numerous plant species from Limpopo Province that were investigated to identify novel plant extracts that could be used as alternatives to conventional cattle acaricides against ticks. Plant species such as *Carica papaya*, *Tagetes minuta*, *Diospyros lycioides*, *Clerodendrum glabrum*, *Terminalia sericea*, *Calpurnia aurea, Lantana camara*, and *Bulbine latifolia* were cited. In addition, certain medicinal plants with acaricidal properties against cattle ticks are expected to become viable alternatives for controlling ticks in the future. Nonetheless, some researchers have expressed concerns about using medicinal plants to control cattle ticks, citing the need for more solid evidence to support their effectiveness. Despite being widely used by farmers globally, ethnoveterinary practices have often been criticized or dismissed. However, other studies (18, 20) have provided scientific evidence that helps identify effective plant extracts under controlled and reproducible conditions. This enables resources to be directed toward developing products based on these promising plant species. For example, medicinal plants such as *Calpurnia aurea* and *Rotheca alabrum* have been tested for biological activities against *Rhipicephalus* species, showing effects similar to those of conventional acaricides (18, 22).

Furthermore, these types of research offer hope and encouragement to rural farmers who continue to rely on indigenous practices for controlling cattle ticks (18–21). In addition, based on the extensive literature review and the ethnoveterinary practices of the rural communities, numerous extracts from the plant species in Limpopo Province were examined to identify novel botanicals that could serve as alternatives to conventional acaricides for cattle tick control (21). Historically, the primary focus of research on plants with acaricidal effects against cattle ticks has been in Vhembe District, Mopani District, and Capricorn District. However, in Sekhukhune District, there is no record of plants being documented for their acaricidal properties. Nonetheless, ethnobotanical practices related to human primary healthcare remain sufficiently documented (2, 23, 24). Therefore, this study was conducted to document the fragility of oral IK regarding the traditional practices of medicinal plants used as alternative acaricides against cattle ticks in Sekhukhune District.

## Materials and methods

### Study area

**Figure 1:**
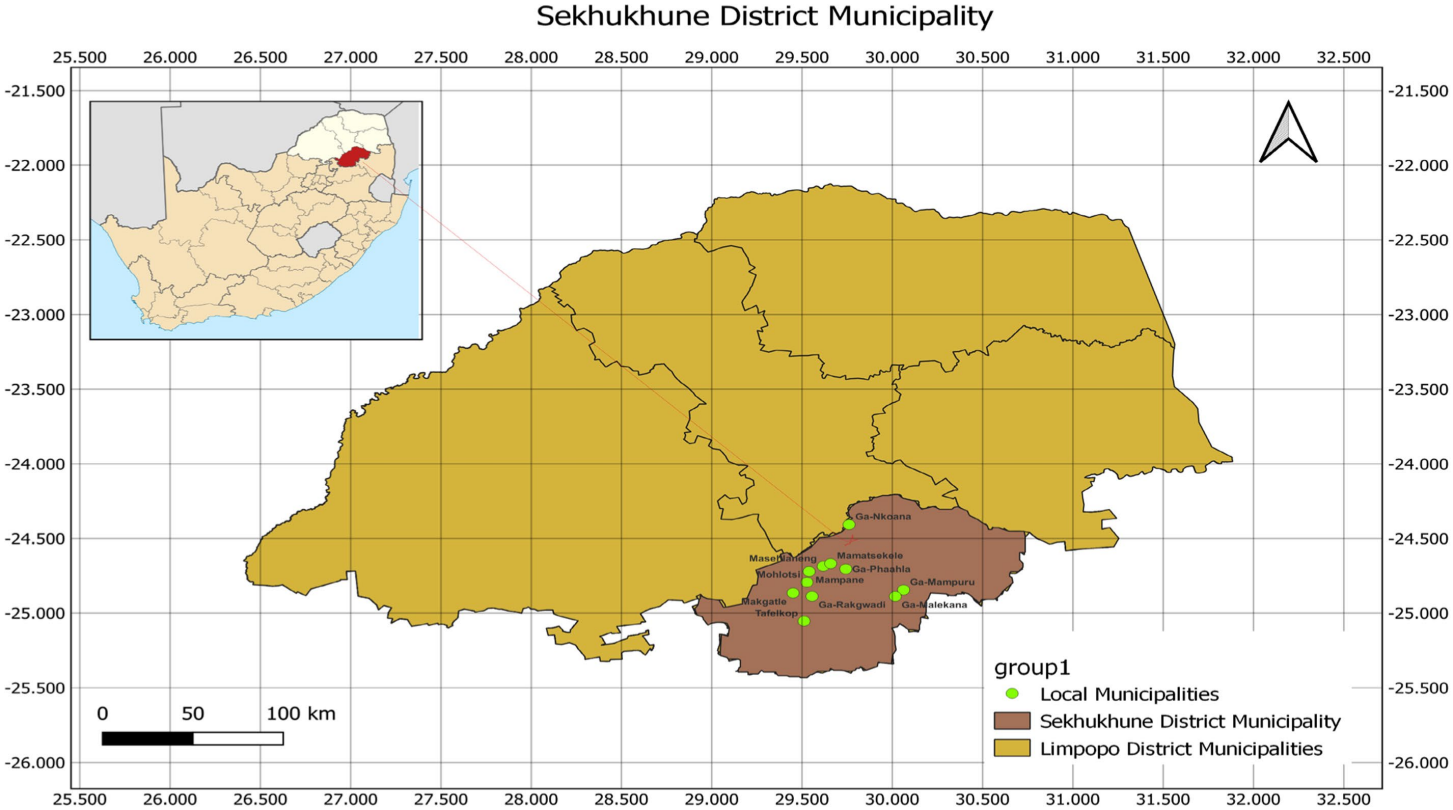
A map of Limpopo Province showing the study area (Sekhukhune District) highlighted in brown color.

The study was conducted in Sekhukhune District Municipality, located in the southeastern part of Limpopo Province, South Africa (24°23′27.52″S and 29°50′06″E), as shown in Figure 1. The district covers approximately 13,528 km^2^ of geographical area and is divided into four local municipalities: Makhuduthamaga, Elias Motsoaledi, Ephraim Mogale, and Fetakgomo Tubatse. The regional climate is subtropical, characterized by warm, moist summers and cool, dry winters (23).

The rainfall experienced in the district ranges from 350 mm during the dry season to 650 mm during the wet season, with an average altitude of 494 m above sea level (23, 25). The district is largely rural, with the majority of the indigenous inhabitants in the study area belonging to the Bapedi ethnic group (26).

### Ethnoveterinary knowledge survey

A purposive sampling method was used to select localities based on the availability of different plant biodiversity, number of livestock, and topography position. The four local municipalities were selected, with specific villages targeted within these local municipalities named: Ga-Phaahla, Ga-Nkoana Masehlaneng, Mamatsekele, Mampane, Ga-Mampuru, Ga-Malekana, Makgatle, Mohlotsi, Ga-Rakgwadi, and Tafelkop. Furthermore, participants were selected based on socio-demography factors, ensuring an equal chance of inclusion for all groups in the sample. Permission to conduct the research and to access the communities was obtained from traditional leaders. The purpose of the research was explained to both the traditional leaders and participants in the local language, Sepedi.

### Indigenous knowledge documentation / data collection

Oral interviews were conducted with approximately 250 participants between July 2021 and June 2022. Data were collected using a semi-structured questionnaire and a guided field survey among the participants. A questionnaire was designed to gather data on the names of plants used for controlling cattle ticks, the source of these plants, the part(s) used, the methods of preparing remedies, and different ways of administering the remedies to cattle. The questionnaire was translated into the local native language to ensure an accurate understanding of the questions asked.

### Plant collection and identification

Plants were collected from their natural habitat in Sekhukhune District, under the guidance of the participants. Plant samples were collected when mentioned twice for ethnoveterinary use by at least two participants (27). Botanical data were collected using a collection form prescribed by the South African National Biodiversity Institute (SANBI). Pictures of the plants were taken with a digital camera, and precise coordinates of the locations were recorded using a GPS instrument. Specimens were collected, labeled, and pressed according to methods described in a previous study (28). One specimen was sent to the SANBI for identification. Approximately 2 kg of fresh plant materials was collected, dried in the shade, ground to a powder, and stored in darkened glass jars for subsequent laboratory investigations.

### Statistical analysis

All collected data were analyzed using statistical software (Stata 17.0). Descriptive statistics were applied for quantitative analysis. Univariate analyses were presented using frequencies and percentages. Bivariate analysis using cross-tabulation was presented using frequency counts and percentages.

## Results

### Demographic information of the participants

A total of 250 participants, with an age range from 18 to over 60 years, were recruited into the study. Among these participants, the most dominant group included the male participants, comprising 60.8%, followed by the female participants at 3%. In addition, the male and female participants in the age group of 60 and above were the most dominant, with a frequency of 34 and 22.0%, respectively. The demographic information of the participants during the study period is provided in Table 1.

**Table 1:** Demographic information of the participants during the study period.

Characteristic of participants	Category	Sex	Frequency (*N* = 250)	Percentage
Sex		Male ⚣	152	60.8
		Female ♀	98	39.2
Age group (years)	18–29	Male ⚣	6	2.4
		Female ♀	1	0.4
	30–39	Male ⚣	17	6.8
		Female ♀	4	1.6
	40–49	Male ⚣	18	7.2
		Female ♀	14	5.6
	50–59	Male ⚣	26	10.4
		Female ♀	24	9.6
	≥ 60	Male ⚣	85	34
		Female ♀	55	22

### Level of education in the study area during the study period

The results of the survey revealed the majority of the participants were either secondary school dropouts or still in secondary school, with the male and female participants comprising 22 and 17.6%, respectively. Moreover, 18.4% of the male participants and 11.2% of the female participants were found to have dropped out of primary school. Among all the participants, 11.2% of the male participants and 3.2% of the female participants were found to either have a tertiary qualification or be studying at the university. Furthermore, 9.2% of the male participants and 7.2% of the female participants were found to have no formal education. Table 2 provides a summary of the level of education during the study period.

**Table 2:** The level of education in the study area during the study period.

Characteristic of participants	Category	Sex	Frequency (*N* = 250)	Percentage
Level of education	No formal education	Male ⚣	23	9.2
		Female ♀	18	7.2
	Primary	Male ⚣	46	18.4
		Female ♀	28	11.2
	Secondary	Male ⚣	55	22
		Female ♀	44	17.6
	Tertiary	Male ⚣	28	11.2
		Female ♀	8	3.2

### An indicative summary of the level of employment in the study area

The survey revealed that a significant proportion (52.8%) of the participants were dependent on social welfare grants, as shown in Table 3. The survey further revealed that 16.8% had professional jobs, while 16% of the participants had non-professional jobs. In addition, 12.8% of the participants were unemployed, with 1.6% having unspecified sources of income.

**Table 3:** An indicative summary of the employment of the participants in the study.

Category		Frequency (*N* = 250)	Percentage
Employed	Professional jobs	42	16.8
	Non-professional jobs	40	16
Unemployed	Social welfare grant	132	52.8
	Unemployed	32	12.8
	Other	4	1.6

### Ethnoveterinary knowledge and practices involving the locally available medicinal plants

The results of the locally available medicinal plants and practices within the study area are shown in Table 4.

**Table 4:** Ethnoveterinary knowledge and practices involving the locally available medicinal plants within the study area.

Family name	Scientific name	Vernacular name	Part used	Preparation and administration
*1. Asphodelaceae*	*Aloe marlothii*	Sekgopha	Leaves	Crushed leaves are mixed with water and applied to infested sites, or infusions are given orally.
	*Aloe castanea Schönland*	Segafane	Leaves	Crushed leaves are mixed with water and applied to infested sites.
*2. Asparagaceae*	*Agave americana*	Sekgokgopha	Leaves	Crushed leaves are mixed with water and applied to infested sites.
	*Asparagus laricinus Burch.*	Leutlwautlwane	Bulbs	Crushed bulbs are mixed with water and applied to infested sites.
*3. Asteraceae*	*Schkuhria pinnata (Lam.) Kuntze ex Thell.*	Shatume	Whole plant	Crushed whole plant is mixed with water and applied to infested sites.
	*Kleinia longiflora DC*	Sebale	Stems	Crushed stems are mixed with water and applied to infested sites.
*4. Araceae*	*Stylochaeton natalensis Schott*	Mokunya	Bulbs	Crushed bulbs are mixed with water and applied to infested sites.
*5. Euphorbiaceae*	*Croton gratissimus Burch.* Var. *gratissimus*	Mologa	Leaves	Crushed leaves are mixed with water and applied to infested sites.
	*Euphorbia cooperi N.E.Br ex A.Berger*	Mokgwakgwata	Stems	Crushed stems are mixed with water and applied to infested sites.
	*Jatropha zeyheri Sond.*	Sefapabadimo	Roots	Crushed roots are mixed with water and applied to infested sites.
	*Tragia dioica*	Motšhetšherepe / motšhegerepe	Leaves	Crushed leaves are mixed with water and applied to infested sites.
*6. Fabaceae*	*Elephanttorrhiza elephantine (Burch.) Skeels*	Mošitšane	Leaves and roots	Crushed leaves and roots are mixed with water and applied to infested sites.
	*Peltophorum africanum Sond.*	Mosehla	Bark	Crushed bark is mixed with water and applied to infested sites.
	*Rhynchosia atropurpurea Germish.*	Tshokang	Leaves	Crushed leaves are mixed with water and applied to infested sites.
	*Senegalia burkei (Benth.) Kyal. & Boatwr.*	Mokgwaripa	Thorns	Pull out ticks
	*Senegalia mellifera (Vahl) Seigel & Ebinger subsp. detinens (Burch.) Kyal. & Boatwr.*	Mongana	Thorns	Pull out ticks
*7. Hyacinthaceae*	*Drimia altissima (L.f) Ker Gawl*	Sekgaga	Bulbs	Crushed bulbs are mixed with water and applied to infested sites.
	*Drimia sanguinea*	Sekanama	Bulbs	Crushed bulbs are mixed with water and applied to infested sites.
	*Albuca shawii Baker*	Serantša	Bulbs	Crushed bulbs are mixed with water and applied to infested sites.
	*Ledebouria inquinata (C.A.Sm.) Jessop.*	Mantsikinyane	Bulbs	Crushed bulbs are mixed with water and applied to infested sites.
*8. Hypoxidaceae*	*Hypoxis obtusa Burch. ex Ker Gawl.*	Monnamaledu	Bulbs	Crushed bulbs are mixed with water and applied to infested sites
*9. Malvaceae*	*Dombeya rotundifolia (Hochst.) Planch. var. rotundifolia*	Mokgoba	Leaves	Crushed leaves are mixed with water and applied to infested sites.
*10. Orchidaceae*	*Eulophia petersii (Rchb.f.) Rchb.f.*	Mongwang wa taba	Bulbs	Crushed bulbs are mixed with water and applied to infested sites.
*11. Passifloraceae*	*Adenia fruticose Burtt Davy subsp. fruticosa*	Mopowane	Bulbs	Crushed bulbs are mixed with water and applied to infested sites.
*12. Rutaceae*	*Verpris reflexa I. Verd*	Pharagobe	Leaves	Crushed leaves are mixed with water and applied to the infested site.
*13. Solanaceae*	*Solanum litchtensteinii Wild*	Thola	Fruits	Juice of the fruits is applied to infested sites. Apply the fruit extract directly to the tick, repeatedly.
*14. Verbenaceae*	*Lippia javanica (Burm.f.) Spreng*	Mošunkwane	Leaves	Crushed leaves are mixed with water and applied to infested sites.
*15. Vitaceae*	*Cissus quadrangularis L.*	Monokelela	Stems	Crushed stems are mixed with water and applied to infested sites. Apply the extract directly to the tick, repeatedly.

The results of the survey revealed 15 different families of the medicinal plants available within the study area. Among the 15 families, *Fabaceae* was found to have five species of medicinal plants. *Euphorbiaceae* and *Hyacinthaceae* were found to have four species, followed by *Asphodelaceae*, *Asparagaceae*, and *Asteraceae, all of* which were found to have two species of medicinal plants.

Furthermore, nine families—namely, *Araceae*, *Hypoxidaeae*, *Malvaceae*, *Orchidaceae*, *Passifloraceae*, *Rutaceae*, *Solanaceae*, *Verbenaceae*, and *Vitaceae—*were found to have only one species of a medicinal plant. The results further revealed that the majority of the plants were either administered orally or topically in the form of liquid infusions and pastes, respectively. Furthermore, *Cissus quadrangularis* was the most frequently cited species. *Cissus quadrangularis* is widely distributed in the district.

### Use of the different plant parts in the preparation of ethnoveterinary medicine

**Figure 2:**
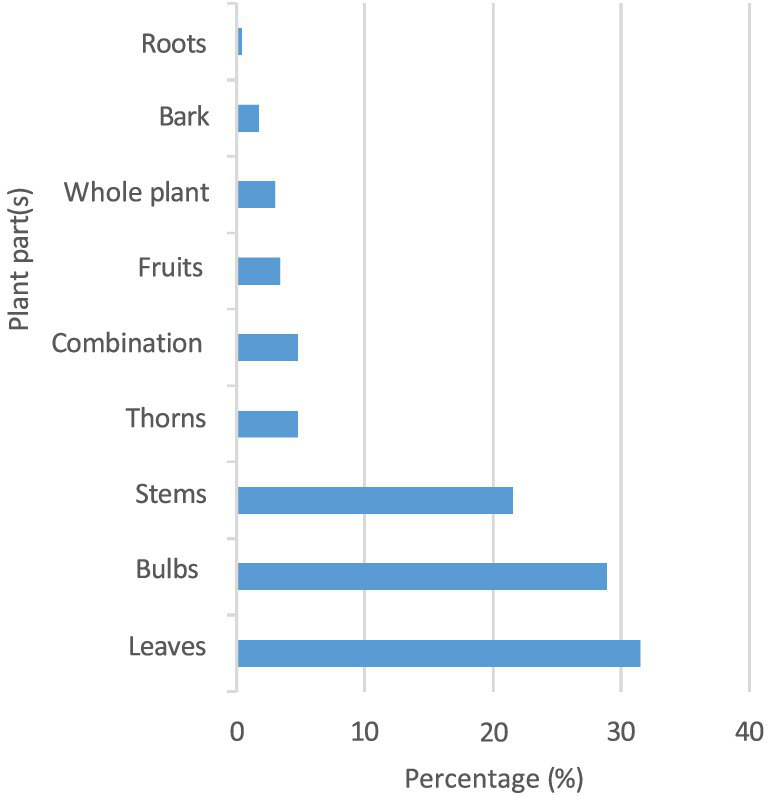
Plant parts used in the preparation of ethnoveterinary medicine within the study area.

Figure 2 shows the plant parts used in the ethnoveterinary practices within the study area. The results of the survey revealed that the leaves emerged as the predominant part used in preparing EVM, accounting for 31.47%, followed by a combination of medicinal plants (4.74%) and the whole plant (3.02%). The roots were the least used part, accounting for 0.43%.

### Different methods for controlling cattle ticks in the study area

Table 5 shows a summary of the methods used to control cattle ticks within the study area. The results revealed that the majority of the participants used a combination of ethnoveterinary medicine and conventional acaricides, with 33.2% of the male participants and 22% the female participants, followed by conventional acaricides, with 19.6% of the male participants and 13.2% of the female participants. The least common method was EVM, with 8% of the male participants and 4% of the female participants.

**Table 5:** Different methods for controlling cattle ticks in the study area.

	Category	Sex	Frequency (*N* = 250)	Percentage
Method of animal treatment	Ethnoveterinary medicine	Male ⚣	20	8
		Female ♀	10	4
	Conventional acaricides	Male ⚣	49	19.6
		Female ♀	33	13.2
	A combination of ethnoveterinary medicine and conventional acaricides	Male ⚣	83	33.2
		Female ♀	55	22

### Comparison of the age and different methods for controlling cattle ticks

Table 6 shows a summary of the methods used for controlling cattle ticks in comparison with the age groups within the study area. The results revealed that the participants shared one commonality: regarding all the methods of controlling ticks, the older participants, aged 60 and above, were more knowledgeable compared to other age groups. This may be due to the perception among the youth that indigenous knowledge is outdated.

**Table 6:** Comparison of the age and different methods for controlling cattle ticks.

	Category	Age	Frequency (*N* = 250)	Percentage
Comparison of the age and different methods for controlling cattle ticks	Ethnoveterinary medicine	18–29	0	0
		30–39	3	1.2
		40–49	0	0
		50–59	7	2.8
		>60	20	8
	Conventional acaricides	18–29	3	1.2
		30–39	7	2.8
		40–49	10	4
		50–59	18	7.2
		>60	44	17.6
	A combination of ethnoveterinary medicine and conventional acaricides	18–29	4	1.6
		30–39	11	4.4
		40–49	22	8.8
		50–59	25	10
		>60	76	30.4

Nonetheless, as shown in Table 6, the younger generation had limited knowledge about all the methods, with 0% of them having knowledge about EVM, 1.2% about conventional acaricides, and 1.6% about the combination of ethnoveterinary medicine and conventional acaricides.

## Discussion

### Demographic information of the participants

Ethnoveterinary practice is a gender-based activity that requires a substantial amount of skills, knowledge, natural resources, and traditional beliefs, with men, women, and children of the household involved in rearing and practicing indigenous primary healthcare of livestock. The ethnic groups inhabiting Sekhukhune District use diverse flora in controlling tick infestations in cattle. Generally, the practice of EVK is mostly limited to the elders of the communities (16, 29, 30). Nonetheless, the participants in the research demonstrated rich EVK regarding the practices of tick control in cattle. As shown in Table 1, the majority of the male participants were the most knowledgeable, with approximately 60.8%, and this observation has also been reported by previous studies (17, 30–32). In addition, it was observed that the local male participants were mostly subjected to inherited livestock ownership from their parents. This, in turn, created a gender-biased system rooted in local rules that disadvantaged female individuals.

Furthermore, male individuals, starting from the age of 18, are taught ethnoveterinary practices as primary healthcare of cattle by the older generation through passive oral communication and practical applications. They (young male individuals) exchange EVK among peers at the dipping station or during herding in the bushes (16). This provided male individuals with significantly greater control over ethnoveterinary knowledge as compared to their female counterparts (16).

Moreover, sociocultural norms in many settings may limit female individuals in cattle care giving (33–35). As shown in Table 1, approximately 39.2% of the female participants were knowledgeable as female individuals are typically subjected to household duties and caregiving for poultry and small-stock (small ruminants). Furthermore, during the interviews, it was observed that the majority of the female participants preferred to let the male participants (old or young) speak as they believed that the male participants had better knowledge of this aspect of tick control in cattle. Moreover, some misconceptions contributed to the limitation of the female participants in cattle caregiving, such as female individuals are not permitted to enter the kraal. Collectively, these findings are supported by those of previous studies (36, 37), which concluded that female individuals have practically minimal knowledge of ethnoveterinary practices.

Currently, female individuals partake in the practices since the majority of households are headed by them. The results of the research correspond with those of a previous study (38). The age group of the knowledgeable participants ranged from 18 to above 60 years, as shown in Table 1. Approximately 2.4% of the participants were young male individuals aged 18–29 years.

They were particularly knowledgeable about the aspect of tick control in cattle and other aspects of primary healthcare in livestock production as they were shepherds. However, only 0.4% of the female participants were knowledgeable. In addition, in the age groups 30–39 years and 40–49 years, 11% of the male participants and 10% of the female participants and 18% of the male participants and 14% of the female participants, respectively, were knowledgeable.

The percentage of the overall knowledgeable participants between the ages of 18 and 49 years was alarming since approximately 90% of the population inhabiting Sekhukhune District is younger than 60 years (39). Furthermore, in the age groups 50–59 years and 60 years and above, 26% of the male participants and 24% of the female participants and 85% of the male participants and 55% of the female participants, respectively, were knowledgeable. Moreover, the oral transfer of IK is under great threat if not conveyed to the right person in time as valuable knowledge is lost with the passing of an elder who carries it (40).

The majority of the youth showed no interest in the indigenous practices for animal health caregiving. Nonetheless, adults and elders regularly practice it and are willing to pass on the IK. Similar results were reported by previous studies (40, 41). It was observed that the older participants believed that EVM is more effective compared to conventional acaricides (7). Furthermore, they supported their practice stating that EVM has fewer side effects.

### Level of education in the study area during the study period

A small percentage of the participants, approximately 16.4% (male and female), lacked formal education, predominantly within the elderly group, as shown in Table 2. They were observed to rely more on IK compared to those with formal education. These findings are consistent with the results reported by a previous study (42). Approximately 83.6% (male and female) of the participants had formal education, ranging from primary to tertiary level. Furthermore, it was observed that the selected knowledgeable participants with formal education and access to additional finance tend not to practice the indigenous traditions, and this might have an influence on the abandonment of ethnoveterinary practices (43). In addition, the diminishing of ethnoveterinary knowledge and practices might be a reflection of cultural changes observed in migration or contact with other cultures, changes in the environmental resources and beliefs, and technology interventions. Furthermore, with access to modernized practices, the younger generation tends to use veterinary services and over-the-counter conventional acaricides, neglecting indigenous practices. Thus, the results are supported by those of previous studies (16, 20, 44). Furthermore, employing modernized practices in treating or controlling ticks in cattle production posed challenges for the participants. When utilizing conventional medicine, issues such as skin burns and the development of acaricidal resistance in ticks were observed, particularly with prolonged use, similar results were reported by a previous study (45).

### An indicative summary of the level of employment in the study area

The primary household income source was social welfare grants, accounting for approximately 52.8% (male and female), as shown in Table 3. The results of this study agree with those of a previous study (38), which reported that the majority of participants were of the older generation (retirement age). However, these results are in contradiction with those of another study (43), which reported that the majority of participants who used medicinal plants to control parasites in livestock were unemployed; nonetheless, they were elders. This could be attributed to the fact that a significant number of youth and adults might have been occupied with work or school during the day when the interviews were conducted or they might have migrated to urban areas for new opportunities (20). This was followed by the participants in professional jobs (16.8%), non-professionals (16%), unemployed (12.8%), and the smallest group, other (1.6%), which included varsity students. It was observed that the majority of the participants were dependent on government social welfare grants. The participants, therefore, used EMV to provide their livestock with basic healthcare (42).

### Ethnoveterinary knowledge and practices involving the locally available medicinal plants within the study area

All plant species referenced in Table 4 were collected from the wild as it is believed that cultivated plants, those near roadsides, or those within homesteads are not as effective as EVM, contrary to the results reported by a study (46), which stated that selected EVM were found on farms. The results of this study revealed a total of 28 plant species from 15 families cited by the participants. *Fabaceae* emerged as the dominant family with five plants, consistent with the results reported by previous studies (20, 32, 47), followed by *Euphorbiaceae* and *Hyacinthaceae* (four plant species each), *Asparagaceae*, *Asphodelaceae,* and *Asteraceae* (two plant species each), and *Araceae*, Hypoxidaceae, *Malvaceae*, *Orchidaceae*, *Pascifloraceae*, *Rutaceae*, *Solanaceae*, *Verbenaceae,* and *Vitaceae,* each with one plant species cited. However, contrary to the results reported by a previous study (48), which stated that *Lamiaceae* is the dominant family used in EVM, this discrepancy might be due to differences in species abundance and a lack of IK in the location. The majority of the medicinal plants used in controlling cattle ticks were applied as water-based extracts, followed by raw juice and roasted formulations. However, the roasted plant parts were mixed with paraffin to create a paste, as detailed in Table 4. The primary route of administration was topical, followed by oral. Moreover, *Aloe Marlothii* was the only medicinal plant that was used in both forms of administration, similar to the results reported by previous studies (11, 14, 49).

The study identified some selected medicinal plants with precautionary warnings due to skin irritation, while the plants producing milky latex or fruits were deemed highly poisonous. Some selected medicinal plants had thorns that required careful handling. Furthermore, species such as *Euphorbia cooperi* are known for their highly poisonous milky latex to humans and animals. According to previous studies (50–52), *Euphoria cooperi* may have therapeutic potential against inflammatory diseases and possess antioxidant activity. In addition, other studies (53, 54) have reported that *Solanum lichtensteinii* might have therapeutic potential for skin-related diseases. Nevertheless, the majority of the cited medicinal plants in the study are user-friendly and commonly used in human healthcare by the Bapedi ethnic group in Limpopo Province, including *Lippia javanica*, *Schkuhria pinnata*, *Aloe marlothii*, *Kleinia longiflora,* and *Vepris reflexa* (12, 24, 55, 56).

### Use of the different plant parts used in the preparation of ethnoveterinary medicine within the study area

The cited plant parts used in ethnoveterinary practices are shown in Figure 2. The leaves were the most commonly used part to prepare medication (31.47%), followed by the bulbs (28.88%), the stems (21.55%), the thorns, a combination of medicinal plants (4.74%), the fruits (3.45%), the whole plant (3.02%), the barks (1.72%), and the least used plant part, the roots (0.43%). According to the participants, the leaves are the most commonly used medicinal plant part in EVM applications due to their availability and abundance. Moreover, they are efficient and carry the least risk of damaging the plant. However, the roots were the least used plant part in the study, which might be attributed to the fact that the roots are not easily accessible and their use could endanger the plant species.

According to previous studies (18, 49, 57–61), which reported similar results, the leaves of selected medicinal plants were found to possess acaricidal activity against cattle ticks. Furthermore, other studies recorded that the following plant parts possessed acaricidal activity and mechanisms for controlling ticks: bulbs, stems, bark, roots, thorns, fruits, and the whole plant. The results are supported by those of previous studies (16, 40, 60, 62). However, in this study, seeds were not cited by the participants. Nevertheless, a previous study (61) reported that cumin seeds (*Cuminum cyminum*) possess acaricidal activity against cattle ticks. This information was not cited in the study due to the origin or availability of the plant in the study area. The plant parts were collected from the wild and used fresh, with no reference to storing them for future use. However, a few participants reported that *Stylochaeton natalensis* and *Hypoxis obtuse* were scarcer in their areas, so they tended to store the plant parts. Moreover, the predominance of fresh materials in ethnoveterinary medicine is contrary to human traditional medicine. This is explained by the fact that the participants resort to EVM only when remedies are needed for their animals. The plant parts were prepared as both monotherapy and in combination. In addition, EVM was applied when ticks were noticeable, and oral administration was practiced daily.

### Different methods for controlling cattle ticks in the study area

The results revealed that the participants in the study used a combination of EVM and conventional acaricidal methods, with 33.2% of the male participants and 22% of the female participants. This was followed by conventional acaricidal methods, with 19.4% of the male participants and 13.2% of the female participants. The least used method was EVM, with 8% of the male participants and 4% of the female participants, as shown in Table 5. Similar results were reported by a study (63), which stated that in South Africa, indigenous people preferred to use traditional medicine even when conventional medicine was available.

However, the literature indicates that using conventional acaricides for tick control is a widely used method (63, 64). It has several advantages, such as being applicable over a large population with minimal lag time. Moreover, in the district selected, the dipping stations are supplied with conventional acaricides by the government for controlling cattle ticks. Eraditick emerged as the dominant conventional acaricidal method (39%), followed by a combination of conventional acaricides (22%), Deadline (21%), Taktic (10%), Supona aerosol (1.6%), Triatix, Copper supadip, Albotic, Delta dip, and Dazzel NF (1.2%), and the least used conventional acaricidal method was Pro-dip cyperthrin (0.81%).

### Comparison of the age and different methods for controlling cattle ticks

As shown in Table 6, the results revealed that a combination of EVM and conventional acaricides was most commonly used by the elderly generation (60 years and above), with a frequency of 30.4%, followed by conventional acaricides (17.6%), and the least used method was EVM (8%). Furthermore, the other age groups were less knowledgeable. However, this result is in contrast with those of a previous study (20), which reported that the majority of the conventional acaricides were used by the youth as they believed that conventional acaricides were more convenient and required less work. The study also reported that ethnoveterinary knowledge resources, such as water and transport, were less utilized unless they used pour-on. In addition, when conventional acaricides are used for prolonged periods, ticks develop resistance to them and the compounds are not easily leashed in the environment. These results are supported by those of previous studies (45, 65).

## Conclusion

The four selected local municipalities within Sekhukhune District are predominantly rural, with the majority of the population being Bapedi. Inhabitants of these municipalities possess extensive knowledge of ethnoveterinary practices for controlling cattle ticks and other essential healthcare practices for livestock. Based on the reported results, 28 medicinal plants have been identified as possessing acaricidal activity, underscoring the continued importance of ethnoveterinary practices in livestock farming.

Ethnoveterinary knowledge (EVK) is primarily restricted to herders, farmers, and elderly men in the communities. Although elderly women exhibit moderate knowledge, the majority of the youth remain unaware of these indigenous practices due to modernization trends. Furthermore, given that the majority of the knowledgeable participants were elderly, there is a risk to the preservation of EVK.

Therefore, the study aimed to conserve traditional EVK from extinction and document its knowledge in its purest form. Furthermore, ethnoveterinary practices reflect the unique cultural and ecological context of the region, underlining the need for continued research, documentation, and potential incorporation of these methods into broader tick control strategies to address the challenges posed by chemical resistance and the environmental impacts of conventional acaricides.

## Figures and Tables

**Figure A1:**
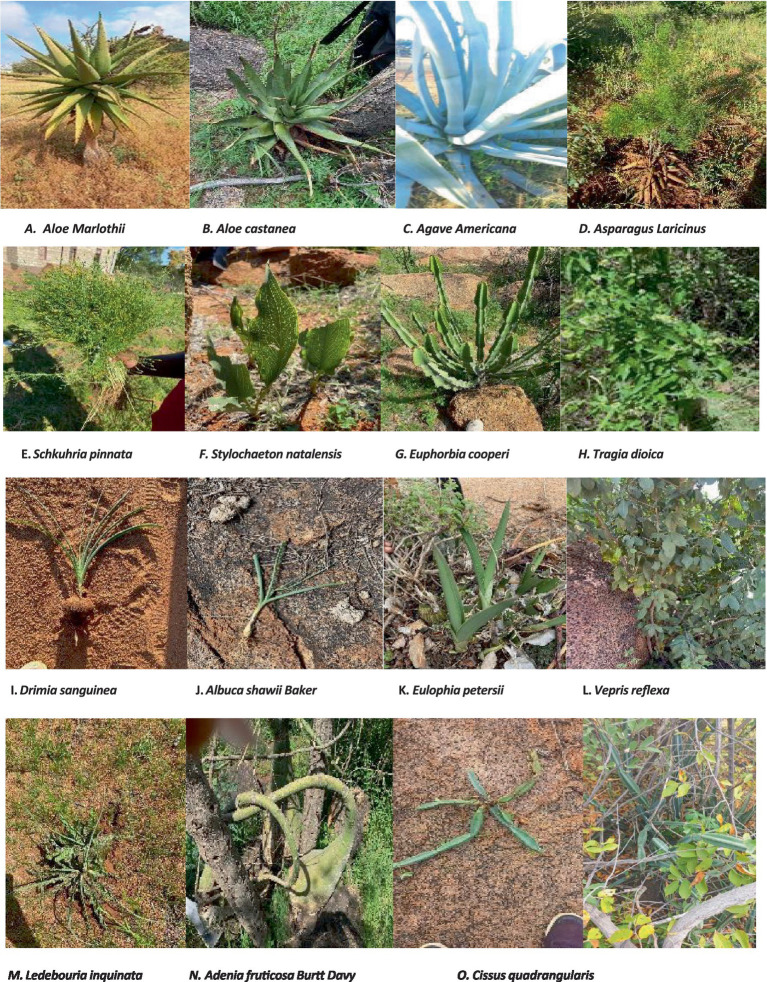
Selected medicinal plants available within the study area.

## Data Availability

The datasets presented in this study can be found in online repositories. The names of the repository/repositories and accession number(s) can be found in the article/supplementary material.
